# Surging trends in prescriptions and costs of antidepressants in England amid COVID-19

**DOI:** 10.1007/s40199-021-00390-z

**Published:** 2021-03-13

**Authors:** Shahad A. Rabeea, Hamid A. Merchant, Muhammad Umair Khan, Chia Siang Kow, Syed Shahzad Hasan

**Affiliations:** 1 Department of Pharmacy, School of Applied Sciences, University of Huddersfield, Huddersfield, United Kingdom; 2 Department of Pharmacy Practice, Faculty of Pharmacy, Ziauddin University, Karachi, Pakistan; 3 School of Postgraduate Studies, International Medical University, Kuala Lumpur, Malaysia

**Keywords:** Mental health, Interventions, Antidepressants, Anxiety, Depression, SARS-CoV-2, Prescribing trends, Cost

## Abstract

The social restrictions amid coronavirus disease 2019 (COVID-19) pandemic have posed a serious threat to mental health and have implications in the use of medications for mental health including antidepressants (ADs). This study investigated the trends in prescriptions and costs of various ADs in England during COVID-19 pandemic. National prescribing rates and net ingredient costs (NIC) of all ADs prescriptions during 2016 to 2020 were analyed. The total number of ADs prescriptions dispensed during COVID-19 pandemic (January to December 2020) were 78 million, 4 million more than in 2019 that costed NHS England £ 139 million more than in 2019. Sertraline, an SSRI antidepressant drug, alone accounted for an extra £113 million during 2020 than in 2019. The peak dispensing for ADs was observed in March 2020 while the total costs for AD drugs peaked in April 2020. The rising prescription costs for ADs during COVID-19 pandemic is a potential cause of concern, in particular the increasing use in adolescents and younger adults needs attention, who are at a higher risk of life-threatening adverse drug reactions.

**Supplementary Information:**

The online version contains supplementary material available at 10.1007/s40199-021-00390-z.

## Introduction

The coronavirus disease 2019 (COVID-19) pandemic has deeply affected the social, physical, and mental well-being of individuals [1]. Since the emergence of COVID-19 pandemic, there had been a substantial increase in mental health problems worldwide [1]. The COVID-19 pandemic had resulted in the loss of normal life and lack of access to favorable and physical activities, combined with the risk of long-term social isolation. Indeed, loneliness was among the most common feelings experienced by the public during the pandemic and associated lockdowns [2]. These issues are particularly challenging for those living with mental health problems including major depressive disorder [1]. Therefore, it came as no surprise that the pandemic has had a particularly significant impact on peoples already living with depression or those at a high risk of developing depression [3].

Evidence suggests that the prevalence of depression had increased 3-folds in the United States since the occurrence of COVID-19 pandemic [4]. A recent survey conducted in the United Kingdom found that the number of individuals living with depression had almost doubled during the pandemic [5]. Given the significant rise in the prevalence of depression associated with COVID-19, the consumption of antidepressants (ADs) had also increased worldwide [4, 6]. The sharp rise in the consumption of ADs is a major concern given the limited evidence on long-term effectiveness and safety of ADs. Given the inconclusiveness of the findings generated from the clinical trials involving the use of ADs, it is important to investigate the real-world prescribing trends that can provide invaluable information to the practitioners and policy makers to help optimising the use of ADs, particularly in a pandemic backdrop which is likely to have long-lasting implications on public health. 

## Methods

This study examined ADs prescription trends during pandemic (Jan to Aug 2020) in comparison to a similar period during last three years before pandemic (2016–2019) to assess the impact of COVID-19 pandemic and associated lockdowns on AD prescribing. We also assessed the economic impact of the change in prescribing trends represented by the changes in the cost incurred to National Health Service (NHS) England. The analysis included overall prescribing trends of ADs regardless of their indication: unipolar depression, bipolar depression, and off-label uses, amongst others. We also performed a sub-group analysis to study the trends of different classes of ADs: selective serotonin reuptake inhibitors (SSRIs), tricyclic antidepressants (TCAs), serotonin-norepinephrine reuptake inhibitors (SNRIs), and atypical ADs.

The prescribing data of ADs were obtained from the Prescription Cost Analysis (PCA) database, which comprise the numbers of prescription items of all prescriptions dispensed in the community in England [7]. NHS Digital publishes these monthly prescribing datasets from the NHS Business Services Authority (NHSBSA) [7]. The PCA database included standard quantity units, items dispensed, costs, the number of total units dispensed (e.g. tablets, capsules, millilitres) specific to each strength and the brand and net ingredient cost (NIC) – the cost of the medicines as outlined by the drug tariff, or manufacturer or wholesaler (where appropriate) [7, 8]. The database is internally audited to 99% accuracy that is atleast 99% of prescriptions are recorded accurately [9]. The data was analysed in Microsoft Excel to study the prescribing and cost trends for COVID-19 pandemic and pre-pandemic periods. Table S1 provides information on total ADs items and costs vs. sertraline available in England.**.**

## Results

**Fig. 1:**
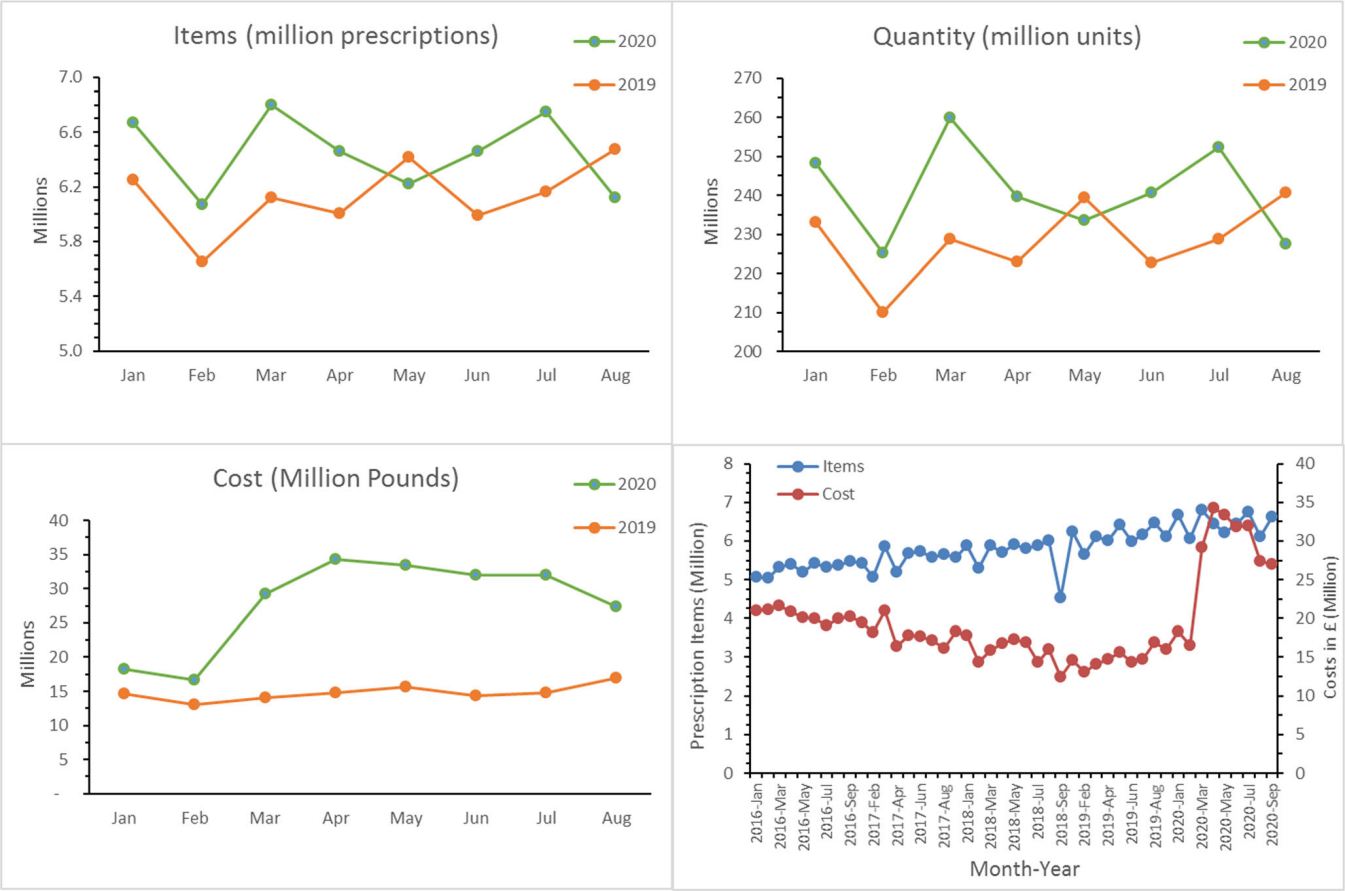
Total antidepressant drugs dispensed and associated cost during COVID-19 pandemic (2020) and preceding years

Figure 1 depicts graphically the prescribing trends of ADs and the associated costs. It was found that the peak number of ADs was dispensed in March 2020 when COVID-19 was officially declared as a pandemic by the World Health Organization. The overall consumption of ADs was higher throughout the pandemic (January 2020 to September 2020), except for May and August, compared to the consumption in similar months during 2019. There were additional 92 million units of ADs dispensed during the pandemic (January 2020 to August 2020) as compared to a similar period last year (Jan to Aug 2019). The associated costs of ADs were also significantly higher in 2020 compared to 2019. A sharp increase in the cost of ADs was noted when the pandemic was at its peak in the United Kingdom with surge in April 2020 to £35 million; this was more than double the cost recorded in the same period in 2019. Shockingly, the total number of ADs prescribed during January 2020 to September 2020 costed NHS over £96 million more than the cost incurred during a similar period in 2019 (Table 1). Albeit a steady historic increase in total AD items from ~5 million items per month in early 2016 to ~6 million items per month in late 2019, the overall cost of AD still maintained a decreasing trend, i.e., ~£21 million per month in early 2016 reduced to £16 million in late 2019. Surprisingly, the cost was increased again during COVID-19, peaking at ~£35 million per month in April 2020.

**Table 1:** Prescription items, quantity dispensed, and costs of antidepressant drugs in 2019 and 2020

Drug class	Items dispensed (Million)		Quantity dispensed (Million)		Costs in £ (Million)	
	Jan-Aug 19	Jan–Aug 20	Jan-Aug 19	Jan–Aug 20	Jan-Aug 19	Jan–Aug 20
Total (BNF) drugs	736.56	738.80	58,238.25	60,282.92	5918.71	6271.22
Total antidepressants	49.33	51.55	1834.90	1926.86	126.66	223.12
Generic products	47.39	49.90	1769.39	1873.76	108.64	220.84
Branded products	1.94	1.86	65.18	61.05	18.02	19.08

The number and quantity of ADs dispensed and costs of generic products of ADs were higher in 2020 compared to 2019. Although the number and quantity dispensed for the branded products of ADs were less (80,000 fewer items and 4 million less quantity) in 2020 compared to 2019, the branded products of ADs still costed an extra £1 million in 2020 (Table 1).

Among different classes of ADs, SSRIs and TCAs were the two most prescribed classes of ADs. The prescription items and quantity dispensed for the three classes of ADs (TCAs, SSRIs, and others) were higher in 2020 compared to 2019 (Table 2). However, the cost of prescribing for all four classes of ADs was higher in 2020 compared to 2019. Interestingly, the cost of prescribing SSRIs was 3 times higher (£128.87 m vs £41.14 m) although accounting for only 6% more units dispensed in 2020 compared to 2019. Sertraline, one of the most prescribed SSRIs drugs, accounted for an extra £113 million during 2020 than 2019 for only 1.79 million items additinally dispensed in 2020 (Table S1) that was mainly attributed to the API (active pharmaceutical ingredient) shortages and a significantly higher cost of generic drugs during pandemic (£108m in 2019 vs. £220m in 2020, Table 1).

**Table 2:** Prescription items, quantity dispensed, and costs of antidepressant drugs

Drug class, n (%)	Prescription items dispensed (Million)		Quantity dispensed (Million)		Costs in £ (Million)	
	Jan-Aug 19	Jan–Aug 20	Jan-Aug 19	Jan–Aug 20	Jan–Aug 19	Jan–Aug 20
Selective serotonin re-uptake inhibitors	26.55	27.82	939.94	995.70	41.14	128.87
Tricyclic and related antidepressant drugs	11.24	11.45	560.33	575.05	41.46	42.53
Monoamine-oxidase inhibitors	0.03	0.01	1.71	0.92	3.64	3.89
Other antidepressant drugs	11.27	12.26	324.43	355.03	32.09	47.23

## Discussion

There had been increasing trend in ADs prescribing globally, with almost 2 to 3 folds increase in consumption over the last few years in Asia, Australia, European countries, such as France and the United Kingdom, and the United States [10–13]. A recent study in the United States showed a 21% increase in the number of ADs, antianxiety, and anti-insomnia prescriptions during the first period of the pandemic between February and March 2020, reaching its peak on 15 March, just after the declaration of COVID-19 as a pandemic by the World Health Organization [14]. This is consistent with our findings; we also found a considerable rise in the prescribing of ADs from February to March 2020. The historic trends in ADs prescribing in England shows an annual increase of ~3 to 4 million prescriptions every year (Table S1). Although this did not change significantly in 2020, our findings suggest that the overall costs associated with ADs in England increased significantly during 2020 compared to 2019, hinting at the potential impact of COVID-19 on the health and mental wellbeing of public in large as also reported elsewhere [15, 16].

Many factors may have contributed to the rise in ADs prescriptions worldwide; the most important factor is the increasing prevalence of depression among the general public which had almost doubled during the COVID-19 pandemic [4, 17]. A study from the United States suggested that the prevalence of depression among adults increased three times during the COVID-19 pandemic compared with the pre-pandemic period [4]. Likewise, the rise in depression symptoms during the COVID-19 pandemic was also reported in the United Kingdom [17]. Similar findings were reported by Pierce et al. in which a deterioration in mental health among the general public in the United Kingdom was found [18]. The increasing number of newly diagnosed cases of depressive disorder, coupled with social isolation, psychological changes, changes in lifestyle associated with COVID-19 pandemic leading to poor quality of life, and temporary suspension of various mental health interventions/services were attributed to the global rise in ADs prescribing, consumption, and associated medication costs.

Various concerns have been raised in the literature about the safety of ADs. Violence, akathisia, and suicidal thoughts are the commonly reported side-effects of ADs, particularly in adolescents and young adults [19]. A review based on the data submitted to the Medicines and Healthcare products Regulatory Agency (MHRA) of the United Kingdom showed an increased rate of suicide during early treatment with ADs, particularly SSRIs [20]. Similarly, a meta-analysis of 100,000 patients using ADs concluded that the risk of suicide doubled in children and adolescents, although there was no similar increase in the risk in adults [21]. These findings are particularly important in the context of COVID-19 pandemic as observational data suggest that young adults, i.e., up to 25 years of age, were impacted by the mental health issues during the pandemic, and hence, were more likely to use ADs [18]. It is, therefore, important to optimize the safe use of ADs, particularly in young adults, not only to help with mental health but also in preventing the associated side-effects that may further increase the morbidity and mortality associated with depression in younger adults.

Certain ADs are known  for causing weight gain. For instance, all the cyclic ADs block histamine receptors and cause increased appetite [22]. Likewise, treatment with SSRIs for long term, as occurred with major depressive disorder, may result in weight gain. The evidence suggests that that paroxetine and fluvoxamine are the most problematic SSRIs with regard to undesirable weight gain: paroxetine leads to weight gain in 6% of patients [23], with a gain of weight ranging from 1.6% to 3.6% of baseline body weight [24, 25], while fluvoxamine leads to weight gain of 2.6% of baseline body weight [24]. On the other hand, among the SNRIs, both duloxetine and venlafaxine are associated with weight gain: weight gain ≥7% of baseline weight occurred in 11% of patients receiving duloxetine (either 80 mg/day or 120 mg/day) [26], whereas the mean weight gain within an average of 18 months with venlafaxine was 7 kg [27]. The weight gain associated with ADs may be undesirable during COVID-19 pandemic since body mass index is a strong independent risk factor for severe course of COVID-19 [28] and obesity itself increases the risk to acquire COVID-19 [29]. Therefore, ADs prescribing of ADs amid COVID-19 should take a personalised approach by considering the patient's body weight.

### Limitations of the study

The nationwide data extracted from PCA database provided unique insights into the ADs prescribing trends across England. However, caution should be exercised while extrapolating these findings acknowledging the limitations of the study. First, the findings of the study draw on the number of prescriptions dispensed; therefore, we cannot be assured if the number of items dispensed was all consumed by the patients. Second, we were not able to provide information about the diagnosis or characteristics of individual patients as PCA only provided a population-level data set. Finally, the prescribing and dispensing trends may be characteristic to England and caution should be exercised before extrapolating these findings to other regions.

## Conclusion

Overall, the ADs prescriptions costs were significantly increased during COVID-19 pandemic compared to the pre-pandemic period with additional 4 million prescription items dispensed in 2020 costing NHS England £139 million more than in 2019. The rise in ADs prescriptions costs during COVID-19 pandemic is a potential cause of concern and highlights an urgent need for mental health interventions in the country and strategies to optimise the use of ADs. Further studies are, however, required to assess the age distribution of ADs prescriptions, particularly a focus on adolescents and young adults who are at a higher risk of experiencing life-threatneing adverse effects.

## Supplementary Information

ESM 1 (DOCX 13.6 kb)

## Data Availability

The data that support the findings of this study are available from NHSBSA (https://www.nhsbsa.nhs.uk).

## Declarations

### Research involving human and animal participants

No human or animal participation.

## References

[1] Qiu J, Shen B, Zhao M, Wang Z, Xie B, Xu Y (2020). A nationwide survey of psychological distress among Chinese people in the COVID-19 epidemic: implications and policy recommendations. Gen Psychiatr.

[2] Mental Health Foundation. 2020. Loneliness during coronavirus. https://www.mentalhealth.org.uk/coronavirus/coping-with-loneliness. Accessed 27 August 2020.

[3] Yao H, Chen JH, Xu YF (2020). Patients with mental health disorders in the COVID-19 epidemic. Lancet Psychiatry.

[4] Ettman CK, Abdalla SM, Cohen GH, Sampson L, Vivier PM, Galea S (2020). Prevalence of depression symptoms in US adults before and during the COVID-19 pandemic. JAMA Netw Open.

[5] Coronavirus and depression in adults, Great Britain: June 2020, 2020. Office for National Statistics. https://www.ons.gov.uk/peoplepopulationandcommunity/wellbeing/articles/coronavirusanddepressioninadultsgreatbritain/june2020. Accessed 28 August 2020.

[6] Read J, Renton J, Harrop C, Geekie J, Dowrick C (2020). A survey of UK general practitioners about depression, antidepressants and withdrawal: implementing the 2019 Public Health England report. Ther Adv Psychopharmacol.

[7] NHS Business Services Authority (NHSBSA). 2020. Prescription Cost Analysis (PCA) data. https://www.nhsbsa.nhs.uk/prescription-data/dispensing-data/prescription-cost-analysis-pca-data. Accessed 1 November 2020.

[8] Curtis HJ, Goldacre B (2018). Open prescribing: normalised data and software tool to research trends in English NHS primary care prescribing 1998–2016. BMJ Open.

[9] NHS Digital Prescription Cost Analysis – 2018 England, 2019. NHS Digital. https://digital.nhs.uk/data-and-information/publications/statistical/prescription-cost-analysis/2018. Accessed 1 November 2020.

[10] Bauer M, Monz BU, Montejo AL, Quail D, Dantchev N, Demyttenaere K, Garcia-Cebrian A, Grassi L, Perahia DG, Reed C, Tylee A (2008). Prescribing patterns of antidepressants in Europe: results from the factors influencing depression endpoints research (FINDER) study. Eur Psychiatry.

[11] Gualano MR, Bert F, Mannocci A, La Torre G, Zeppegno P, Siliquini R (2014). Consumption of antidepressants in Italy: recent trends and their significance for public health. Psychiatr Serv.

[12] Martin-Arias LH, Lobato CT, Ortega S, Velasco A, Carvajal A, del Pozo JG (2010). Trends in the consumption of antidepressants in Castilla y León (Spain). Association between suicide rates and antidepressant drug consumption. Pharmacoepidemiol Drug Saf.

[13] Soleymani F, Taheri F, Roughead E, Nikfar S, Abdollahi M (2018). Pattern of antidepressant utilization and cost in Iran from 2006 to 2013 in comparison with other countries. J Epidemiol Glob Health.

[14] America’s State of Mind Report. 2020. https://www.express-scripts.com/corporate/americas-state-of-mind-report. Accessed 20 August 2020.

[15] Serafini G, Parmigiani B, Amerio A, Aguglia A, Sher L, Amore M (2020). The psychological impact of COVID-19 on the mental health in the general population. QJM..

[16] Xiong J, Lipsitz O, Nasri F, Lui LM, Gill H, Phan L, Chen-Li D, Iacobucci M, Ho R, Majeed A, McIntyre RS (2020). Impact of COVID-19 pandemic on mental health in the general population: a systematic review. J Affect Disord.

[17] Groarke JM, Berry E, Graham-Wisener L, McKenna-Plumley PE, McGlinchey E, Armour C (2020). Loneliness in the UK during the COVID-19 pandemic: cross-sectional results from the COVID-19 psychological wellbeing study. PLoS One.

[18] Pierce M, Hope H, Ford T, Hatch S, Hotopf M, John A, Kontopantelis E, Webb R, Wessely S, McManus S, Abel KM (2020). Mental health before and during the COVID-19 pandemic: a longitudinal probability sample survey of the UK population. Lancet Psychiatry.

[19] Lagerberg T, Molero Y, D’Onofrio BM, de la Cruz LF, Lichtenstein P, Mataix-Cols D, Rück C, Hellner C, Chang Z (2019). Antidepressant prescription patterns and CNS polypharmacy with antidepressants among children, adolescents, and young adults: a population-based study in Sweden. Eur Child Adolesc Psychiatry.

[20] McCain JA (2009). Antidepressants and suicide in adolescents and adults: a public health experiment with unintended consequences?. Pharm Ther.

[21] Sharma T, Guski LS, Freund N, Gøtzsche PC (2016). Suicidality and aggression during antidepressant treatment: systematic review and meta-analyses based on clinical study reports. BMJ.

[22] Kivimäki M, Hamer M, Batty GD, Geddes JR, Tabak AG, Pentti J, Virtanen M, Vahtera J (2010). Antidepressant medication use, weight gain, and risk of type 2 diabetes: a population-based study. Diabetes Care.

[23] Lecrubier Y, Judge R, Collaborative Paroxetine Panic Study Investigators (1997). Long-term evaluation of paroxetine, clomipramine and placebo in panic disorder. Acta Psychiatr Scand.

[24] Fava M, Judge R, Hoog SL, Nilsson ME, Koke SC (2000). Fluoxetine versus sertraline and paroxetine in major depressive disorder: changes in weight with long-term treatment. J Clin Psychiatry.

[25] Maina G, Albert U, Salvi V, Bogetto F (2004). Weight gain during long-term treatment of obsessive-compulsive disorder: a prospective comparison between serotonin reuptake inhibitors. J Clin Psychiatry.

[26] Hudson JI, Wohlreich MM, Kajdasz DK, Mallinckrodt CH, Watkin JG, Martynov OV (2005). Safety and tolerability of duloxetine in the treatment of major depressive disorder: analysis of pooled data from eight placebo-controlled clinical trials. Hum Psychopharmacol.

[27] Uguz F, Sahingoz M, Gungor B, Aksoy F, Askin R (2015). Weight gain and associated factors in patients using newer antidepressant drugs. Gen Hosp Psychiatry.

[28] Du Y, Lv Y, Zha W, Zhou N, Hong X (2020). Association of Body mass index (BMI) with critical COVID-19 and in-hospital mortality: a dose-response meta-analysis. Metabolism.

[29] Popkin BM, Du S, Green WD, Beck MA, Algaith T, Herbst CH, Alsukait RF, Alluhidan M, Alazemi N, Shekar M (2020). Individuals with obesity and COVID-19: a global perspective on the epidemiology and biological relationships. Obes Rev.

