# Supplementary material for: Surging trends in prescriptions and costs of antidepressants in England amid COVID-19
Source: Daru. 2021 Mar 13;29(1):217–21. doi: 10.1007/s40199-021-00390-z (PMC7955799; doi:10.1007/s40199-021-00390-z)
Supplement: Supplementary file 1 — (DOCX 13.6 kb) [file 40199_2021_390_MOESM1_ESM.docx]

**Table S1**: Items dispensed (in million) and costs (in million £) of total antidepressants and sertraline between Jan 2016-Dec 2020

| **Year** | **Total AD items dispensed** | **Total AD Costs** | **Sertraline Items** | **Sertraline - Costs** | **Sertraline % Items** | **Sertraline % Cost** |
| --- | --- | --- | --- | --- | --- | --- |
| 2016 | 64.12 | 246.47 | 11.08 | 18.17 | 17.29 | 7.37 |
| 2017 | 67.00 | 217.90 | 12.79 | 16.25 | 19.08 | 7.46 |
| 2018 | 70.40 | 188.59 | 14.68 | 15.06 | 20.85 | 7.99 |
| 2019 | 74.37 | 188.09 | 16.72 | 24.64 | 22.48 | 13.10 |
| 2020 | 78.49 | 327.73 | 18.46 | 138.18 | 23.52 | 42.16 |

AD=Antidepressants
